# Development and Validation of a Predictive Model for Coronary Artery Disease Using Machine Learning

**DOI:** 10.3389/fcvm.2021.614204

**Published:** 2021-02-02

**Authors:** Chen Wang, Yue Zhao, Bingyu Jin, Xuedong Gan, Bin Liang, Yang Xiang, Xiaokang Zhang, Zhibing Lu, Fang Zheng

**Affiliations:** ^1^Department of Laboratory Medicine, Center for Gene Diagnosis, Zhongnan Hospital of Wuhan University, Wuhan, China; ^2^Department of Cardiology, Zhongnan Hospital of Wuhan University, Wuhan, China

**Keywords:** coronary artery disease, prediction model, machine learning, random forest, primary prevention

## Abstract

Early identification of coronary artery disease (CAD) can prevent the progress of CAD and effectually lower the mortality rate, so we intended to construct and validate a machine learning model to predict the risk of CAD based on conventional risk factors and lab test data. There were 3,112 CAD patients and 3,182 controls enrolled from three centers in China. We compared the baseline and clinical characteristics between two groups. Then, Random Forest algorithm was used to construct a model to predict CAD and the model was assessed by receiver operating characteristic (ROC) curve. In the development cohort, the Random Forest model showed a good AUC 0.948 (95%CI: 0.941–0.954) to identify CAD patients from controls, with a sensitivity of 90%, a specificity of 85.4%, a positive predictive value of 0.863 and a negative predictive value of 0.894. Validation of the model also yielded a favorable discriminatory ability with the AUC, sensitivity, specificity, positive predictive value, and negative predictive value of 0.944 (95%CI: 0.934–0.955), 89.5%, 85.8%, 0.868, and 0.886 in the validation cohort 1, respectively, and 0.940 (95%CI: 0.922–0.960), 79.5%, 94.3%, 0.932, and 0.823 in the validation cohort 2, respectively. An easy-to-use tool that combined 15 indexes to assess the CAD risk was constructed and validated using Random Forest algorithm, which showed favorable predictive capability (http://45.32.120.149:3000/randomforest). Our model is extremely valuable for clinical practice, which will be helpful for the management and primary prevention of CAD patients.

## Introduction

Currently, coronary artery disease (CAD) continues to be the principal cause of worldwide incidence and mortality (1, 2). The main pathogenic mechanism of CAD is atherosclerosis, a complicated and constantly progressing process of chronic inflammation characterized by dysfunction of endothelial cells, cumulative deposition of lipoprotein particles, migration of monocyte and macrophage, proliferation of vascular smooth muscle cells (VSMCs), and ultimately contributes to a narrowing of the vessel that impedes blood supply to the heart (3, 4). The reference standard of CAD diagnosis is invasive coronary angiography, which allows for real-time evaluation of the location and the degree of coronary stenosis, and to decide the most suitable therapy (5). However, its use for population screening has been limited by the demand for specialized catheterization laboratory and the possible radiation exposure (6, 7). Consequently, sensitive, specific, and non-invasive indicators for CAD risk assessment are urgently desirable.

Development and progression of coronary atherosclerosis is modulated by multiple interplays between genetic and environmental factors (8). Consistent and convincing evidence has authenticated a casual correlation between lipoprotein-related lipid contents and cardiovascular disease prevalence (9–11). High level of circulating low-density lipoprotein cholesterol (LDL-C) and triglyceride (TG)-rich lipoproteins were related with high risk of CAD, whereas high level of high-density lipoprotein cholesterol (HDL-C) was correlated with low CAD risk. Furthermore, pedigree studies have demonstrated that triglyceride, LDL-C, and HDL-C concentrations are strongly determined by the individual genetic architecture. For instance, rare variants in the apolipoprotein B (*APOB*) and LDL receptor (*LDLR*) genes and common variants in the apolipoprotein E (*APOE*) gene could increase LDL-C contents and were also correlated with increased susceptibility to CAD (12). In addition to cholesterol, the epidemiological studies have also substantiated other canonical risk factors, such as age, male gender, smoking, alcohol drinking, hypertension, diabetes, and obesity. To devise and improve preventive tactics for CAD, it is indispensable to comprehend and properly calculate the etiological contribution of these risk factors. In this study, we sought to evaluate the predictive value of these traditional risk factors in CAD by machine learning algorithms.

## Materials and Methods

### Study Design and Data Collection

This three-stage case-control study, involving 3,112 CAD patients and 3,182 controls, was retrospectively collected from three clinical centers: the development cohort with 2014 CAD cases and 2018 controls from Wuhan Asia Heart Hospital between March 2014 and October 2016, the validation cohort 1 with 837 CAD cases and 876 controls from Zhongnan Hospital of Wuhan University between January 2016 and December 2017 and the validation cohort 2 with 261 CAD cases and 258 controls from Shandong Provincial Hospital between January 2017 and February 2018. The diagnosis of CAD was determined by coronary angiography that stenosis ≥50% in at least one main coronary artery or their major branches. Patients with other cardiac diseases, autoimmune diseases, systemic diseases, and cancers were excluded. The control groups were non-CAD individuals based on physical examination and medical history evaluations. Traditional CAD risk factors such as age, gender, alcohol drinking, cigarette smoking and histories of hyperlipidemia, hypertension, and type 2 diabetes mellitus (T2DM) and clinical information, including blood pressure, body mass index (BMI), fasting plasma glucose (FPG), total cholesterol (TC), total triglyceride (TG), LDL-C, and HDL-C were retrospectively collected from the database of electronic medical records and laboratory test reports. The study was approved by the Ethics Committees of Wuhan Asia Heart Hospital, Zhongnan Hospital of Wuhan University, and Shandong Provincial Hospital and adhered to the tenets of the Declaration of Helsinki. Informed consent was obtained from all participants.

### Machine Learning Algorithms

Logistic regression is a kind of probabilistic statistical classification model, which can be applied to predict the classification of nominal variable based on certain features. The classification is completed by utilizing the logit function to evaluate the outcome probability. As a supervised machine learning algorithm, support vector machines (SVM) can be fitted for both classification and regression. It first maps the data into a multidimensional feature space constructed by the kernel function, and then determines the optimal hyperplane that partitions the training set by the maximum boundary. Decision trees, one of the easiest kinds of decision model, use a tree structure built by recursive partitioning to simulate the correlations between the features and the potential outcomes. Once the model is created, the resulting structure is shown in a human-readable format. Random forests (RF) are modified bagged trees that randomly select the predictor features to split at each node and incorporate the voting results of many decision trees for classification. It retains the many strengths of the decision tree and exhibits high accurateness in disease diagnosis and risk prediction.

### Statistical Analysis

Qualitative variables were expressed as frequencies with proportions, and the differences between cases and controls were examined by Chi-square test. Quantitative variables were shown as mean with standard deviation (SD) and were assessed for normality distribution by the Kolmogorov-Smirnov test. Independent *t*-test and Mann-Whitney *U*-test were performed to compare two groups of continuous variables with or without normal distribution, respectively. A two-sided *P* < 0.05 was considered to be statistically significant.

Least absolute shrinkage and selection operator (LASSO) regression analysis was applied to identify relatively important features. The logistic regression can be fitted to the data using the glm function with the family argument set to binomial and the summary function was used to check the coefficients and their *p*-values. We use the e1071 package to build linear SVM model since it contains the tune.svm function which can optimize the tuning parameters and kernel functions through cross-validation. To build the classification tree model, we use rpart function from party package and inspect the error per split in order to determine the optimal number of splits in the tree, then prune function was used to prune the tree. Randomforest function from randomForest package was used to build random forest model. The specific and optimal tree was determined by the minimum mean of squared residuals and the number of trees constructed in this model was 178, and three variables were randomly selected to split at each node. The prediction model was established by the four aforementioned machine learning algorithms and the predictive capability was evaluated by the area under the receiver operating characteristic curve (ROC) and the precision-recall curve, using precrec package. For final model, ROCR package was conducted to assess the classification accuracy in the development and validation cohorts. The methods and the interpretation of the results are guaranteed by a machine learning algorithm expert. All data analysis was performed in R software (version 3.6.0).

## Results

### Baseline and Clinical Characteristics of Study Population

In the development cohort, CAD patients had significantly higher age, higher body mass index (BMI), higher proportions of smoking, alcohol drinking, histories of hyperlipidemia, hypertension, and T2DM, higher concentrations of TC, TG, LDL-C, and FPG, and lower level of HDL-C, systolic blood pressure (SBP), and diastolic blood pressure (DBP) comparing with controls (Table 1). In two validation cohorts, the baseline and clinical characteristics of study population were basically similar to that of the development cohort (Table 1).

**Table 1:** Baseline and clinical characteristics of the study cohort.

**Variables**	**Development cohort**			**Validation cohort1**			**Validation cohort2**		
	**Control (*N* = 2,048)**	**CAD (*N* = 2,014)**	***P***	**Control (*N* = 876)**	**CAD (*N* = 837)**	***P***	**Control (*N* = 258)**	**CAD (*N* = 261)**	***P***
Age, year	60.22 ± 9.81	62.56 ± 9.77	<0.001	59.23 ± 11.62	61.97 ± 9.33	<0.001	57.26 ± 9.81	60.68 ± 9.87	<0.001
Male, *n* (%)	1,268 (61.91)	1,230 (61.07)	0.582	536 (61.19)	498 (59.5)	0.475	163 (63.18)	200 (76.63)	0.001
BMI, kg/m^2^	23.8 ± 2.45	25.11 ± 3.71	<0.001	23.69 ± 2.4	25.04 ± 3.68	<0.001	24.11 ± 2.15	25.61 ± 4.37	0.001
Smoking, *n* (%)	573 (27.98)	843 (41.86)	<0.001	240 (27.4)	322 (38.47)	<0.001	58 (22.48)	137 (52.49)	<0.001
Alcohol drinking, *n* (%)	511 (24.95)	555 (27.56)	<0.001	215 (24.54)	231 (27.6)	0.023	56 (21.71)	123 (47.13)	<0.001
Hypertension, *n* (%)	786 (38.38)	1,230 (61.07)	<0.001	365 (41.67)	484 (57.83)	<0.001	86 (33.33)	161 (61.69)	<0.001
T2DM, *n* (%)	521 (25.44)	636 (31.58)	<0.001	220 (25.11)	262 (31.3)	0.004	78 (30.23)	102 (39.08)	0.034
Hyperlipidemia, *n* (%)	477 (23.39)	585 (29.05)	<0.001	199 (22.72)	248 (29.63)	0.001	56 (21.71)	84 (32.18)	0.007
TC, mmol/L	4.59 ± 0.82	4.61 ± 1.09	0.009	4.59 ± 0.85	4.63 ± 1.14	0.016	4.81 ± 0.85	4.47 ± 1.4	<0.001
TG, mmol/L	1.24 ± 0.61	1.7 ± 1.07	<0.001	1.26 ± 0.63	1.74 ± 1.07	<0.001	1.14 ± 0.48	1.96 ± 1.29	<0.001
HDL-C, mmol/L	1.3 ± 0.25	1 ± 0.19	<0.001	1.3 ± 0.25	1.02 ± 0.19	<0.001	1.33 ± 0.28	1 ± 0.19	<0.001
LDL-C, mmol/L	2.69 ± 0.61	2.75 ± 0.87	<0.001	2.7 ± 0.65	2.74 ± 0.86	0.003	2.89 ± 0.74	2.86 ± 1	0.143
SBP, mmHg	139.78 ± 25	132.56 ± 19.68	<0.001	139.65 ± 25.18	131.74 ± 19.09	<0.001	128.76 ± 16.67	132.22 ± 17.25	0.02
DBP, mmHg	85.05 ± 16.94	81.02 ± 11.8	<0.001	85.5 ± 17.83	81.47 ± 11.57	0.005	80.97 ± 11.83	79.62 ± 11.48	0.348
FBG, mmol/L	5.37 ± 1.47	5.85 ± 2.02	<0.001	5.36 ± 1.52	5.86 ± 2.26	<0.001	5.41 ± 0.64	6.48 ± 1.95	<0.001

### Machine Learning Model Evaluation

**Figure 1:**
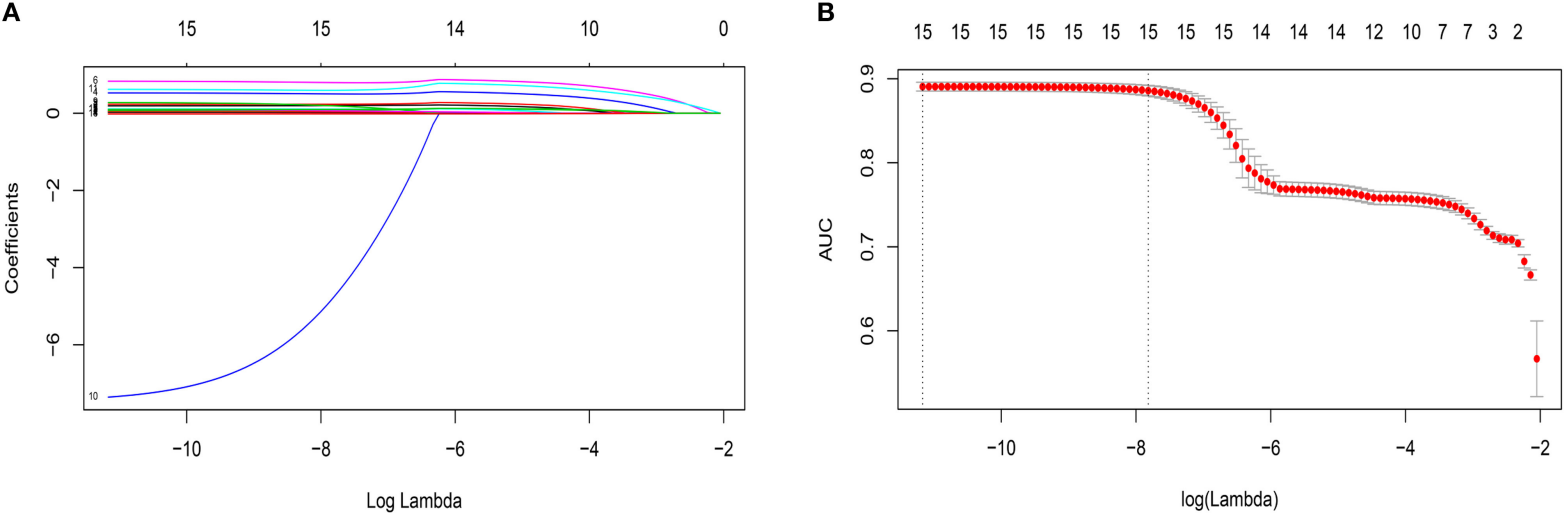
Features selection by least absolute shrinkage and selection operator (LASSO). **(A)** LASSO coefficients profiles (*y*-axis) of the 15 features. The upper *x*-axis is the average numbers of predictors and the lower x-axis is the log(λ). **(B)** Five-fold cross-validation for tuning parameter selection in the LASSO model. The area under the receiver operating characteristic (AUC) with error bar is plotted against log(λ), where λ is the tuning parameter. The dotted vertical lines are drawn at the optimal values by minimum criteria and the one standard error of the minimum criteria (1se criteria). The upper *x*-axis is the average numbers of predictors and the lower *x*-axis is the log(λ). To avoid overfitting, 1se criteria (λ = 0.000402) was selected.

**Figure 2:**
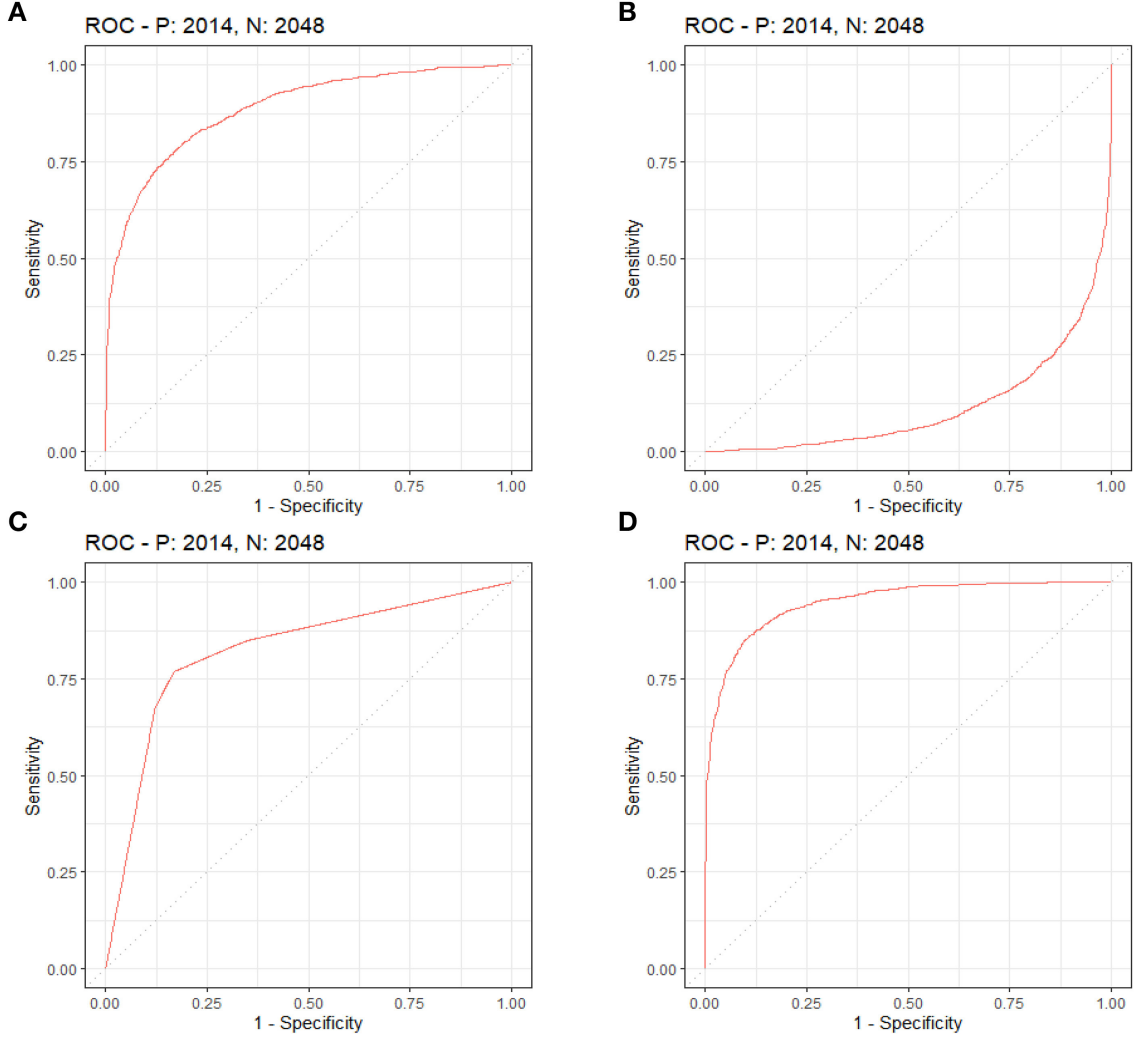
Each machine learning model was assessed by the ROC curve, which plots a curve according to its true positive rate (*y*-axis) against its false positive rate (*x*-axis). The larger area under the curve, the better the prediction accuracy of the model. **(A)** Logistic regression. **(B)** Support vector machine (SVM). **(C)** Decision tree classification. **(D)** Random forest (RF).

**Figure 3:**
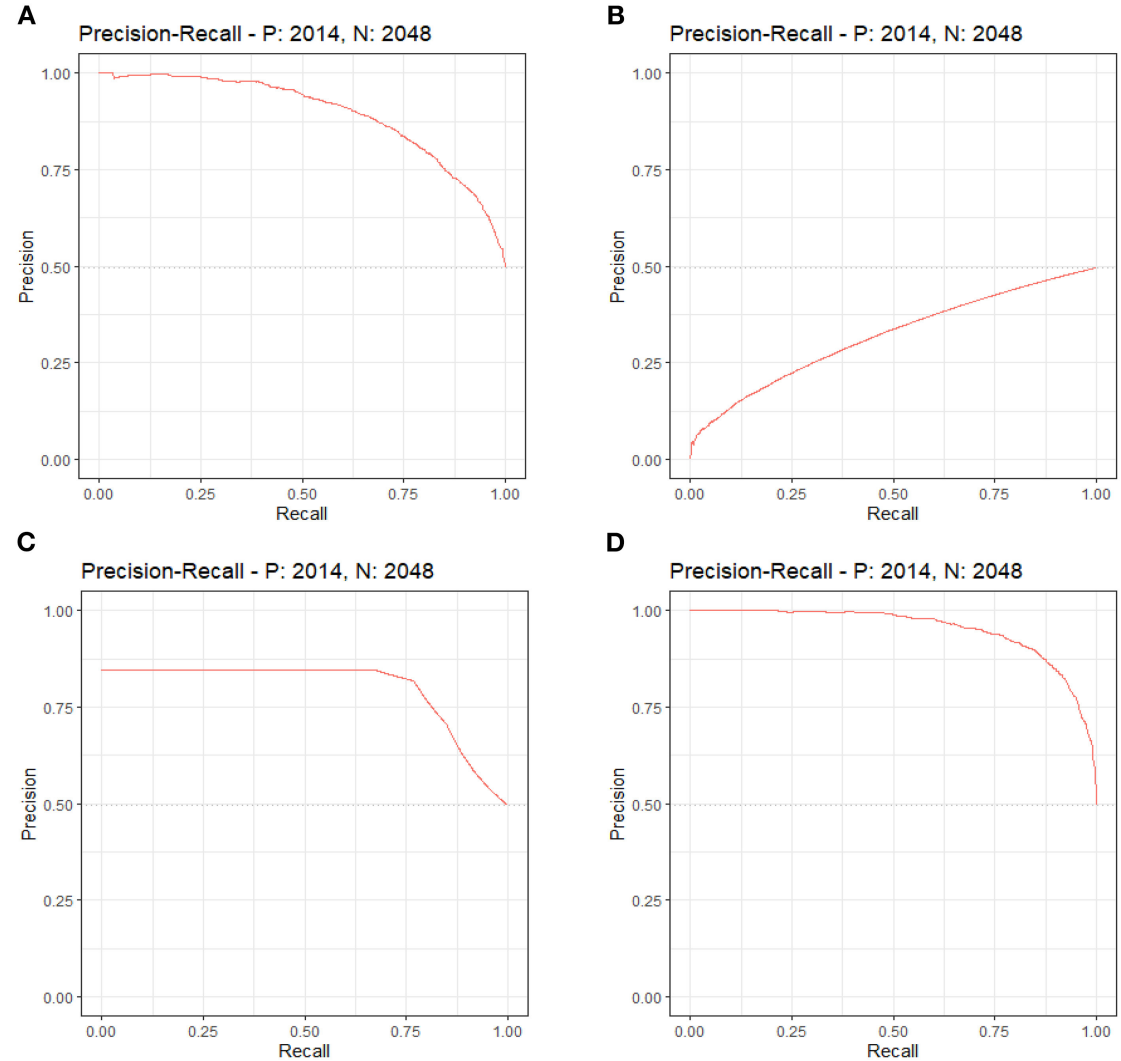
Each machine learning model was evaluated by the precision-recall curve, which displays the trade-off between recall (*x*-axis) and precision (*y*-axis). The bigger area under the curve, the greater predictive capability of the model. **(A)** Logistic regression. **(B)** Support vector machine (SVM). **(C)** Decision tree classification. **(D)** Random forest (RF).

By LASSO regression filtration, all these variables remain significant (Figure 1). Therefore, we utilized four machine learning algorithms (logistic regression, RF, decision tree classification, and SVM) to construct the full model with all these variables, and the ROC curve (Figure 2) and the precision-recall curve (Figure 3) were implemented to assess their performance. Ultimately, random forests model was chosen for further analysis owing to its highest predictive accuracy.

### Model Construction and Validation

**Figure 4:**
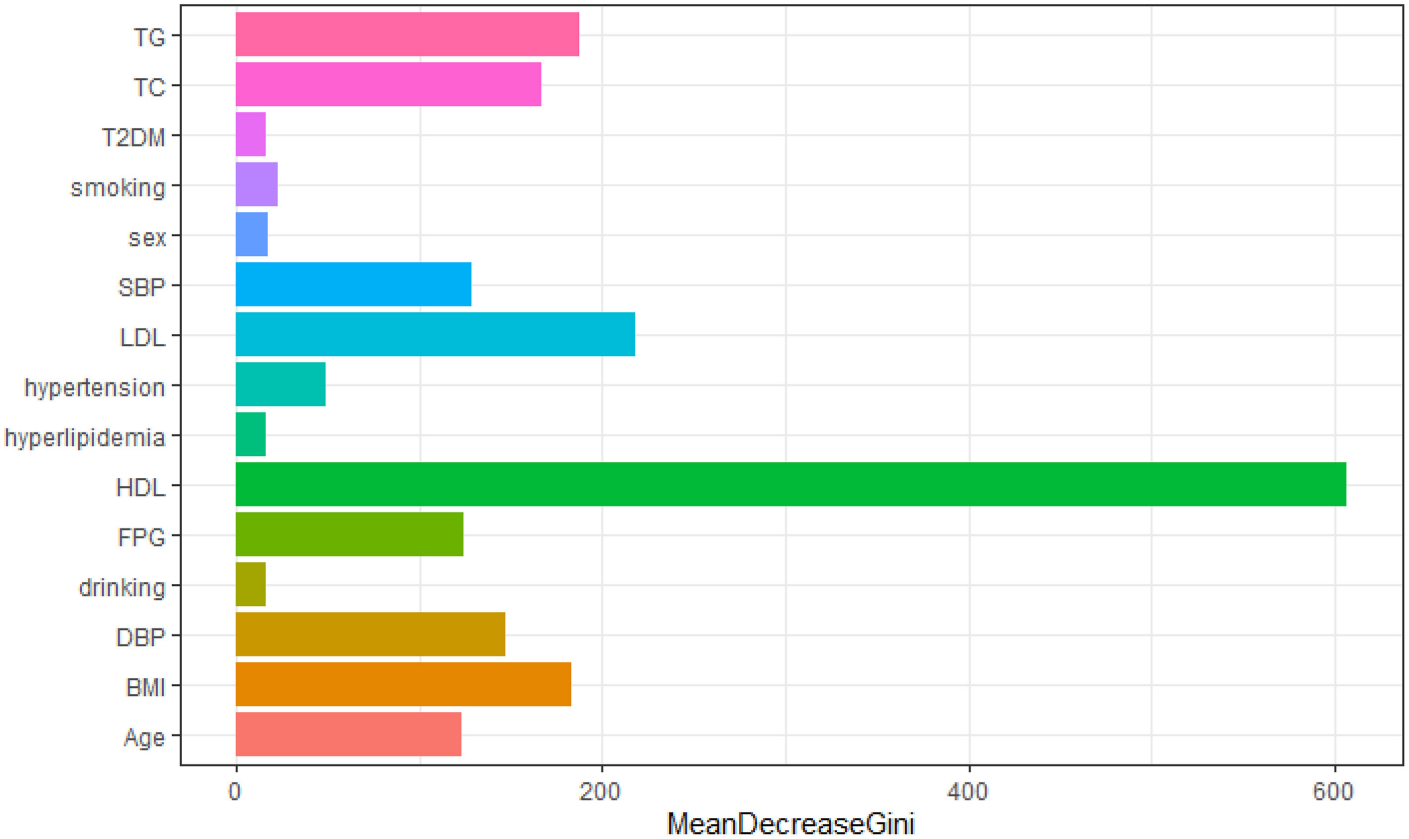
Variable importance plot for the Random Forest model. The *x*-axis is the average decrease in the Gini coefficient, the *y*-axis is 15 risk factors of CAD. The more the average decrease of the Gini coefficient, the more important of the variable.

**Figure 5:**
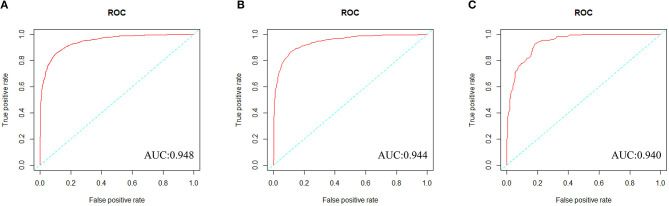
The ROC curves of the Random Forest model in three cohorts. **(A)** To evaluate discrimination performance in the development cohort, area under the curve (AUC) of ROC curve was 0.948. **(B)** To assess the discrimination capability in the validation cohort 1, AUC was 0.944. **(C)** To assess the predictive capability in the validation cohort 2, AUC was 0.940.

We constructed a risk prediction model with all these variables by the random forests. The out-of-bag (OOB) estimate of error rate was 12.26%, indicating that the generalization error of this model is relatively small. The degree of Gini coefficient average decrease implied that HDL-C, followed by LDL-C, TG, BMI, and TC are important features for the risk evaluation of CAD (Figure 4). In the development cohort, this model yielded a high AUC 0.948 (95%CI: 0.941–0.954) to identify CAD patients from controls, with a sensitivity of 90%, a specificity of 85.4%, a positive predictive value of 0.863 and a negative predictive value of 0.894 (Figure 5A). In consistent with the development cohort, favorable discriminatory ability was also demonstrated by two validation cohorts, with an AUC, sensitivity, specificity, positive predictive value, and negative predictive value of 0.944 (95%CI: 0.934–0.955), 89.5%, 85.8%, 0.868, and 0.886 in the validation cohort 1 (Figure 5B), respectively, and 0.940 (95%CI: 0.922–0.960), 79.5%, 94.3%, 0.932, and 0.823 in the validation cohort 2 (Figure 5C), respectively. The model is further shown as a web calculator to facilitate its application (http://45.32.120.149:3000/randomforest).

## Discussion

In the current study, we elucidated the significant contributions of age, gender, alcohol drinking, cigarette smoking, hyperlipidemia, hypertension and T2DM, TC, TG, HDL-C, LDL-C, SBP, DBP, and FBG to the risk of CAD. Subsequently, we constructed and validated a Random Forest model integrated these indexes with a favorable discriminability that can be helpful for the non-invasive identification of CAD patients.

The most important features identified by Random Forest was HDL-C. HDL-C has been regarded as “good cholesterol” largely owing to an inverse association between high HDL-C levels and low CAD risk (13, 14). The main functions of HDL are to facilitate reverse cholesterol transport and regulate inflammation (15). The potential atheroprotective effects of HDL from healthy individuals were remarkably impaired in CAD patients (16, 17). The Framingham study suggested that about 44% CAD clinical events in men with HDL-C >40 mg/dL and near 43% in women with HDL-C >50 mg/dL. In addition, Mendelian randomization genetic studies have suggested that genetically raised HDL-C levels were not correlated with a decreased risk of myocardial infarction comparing with genetic variants associated with lowering LDL-C levels (18). Furthermore, recent large-scale clinical studies had also failed to validate the preventive effect of HDL-C raising treatments on coronary disease (19, 20). These results highlighted that HDL-C levels were not necessarily causally associated with coronary disease and normal serum HDL-C levels did not guarantee free of CAD events (15). Nevertheless, our model illustrated that HDL-C levels made a greatest contribution in CAD risk prediction.

A high serum LDL-C level is a well-established risk factor for cardiovascular disease, especially CAD. Genetic studies have revealed that variants in PSCK9 (proprotein convertase subtilisin/kexin type 9), HMGCR (HMG-coenzyme A reductase), and NPC1L1 (Niemann-Pick C1-like intracellular cholesterol transporter 1) are correlated with decreasing LDL-C levels and low CAD risk (21–23). Moreover, large-scale clinical studies have suggested that decreasing LDL-C by targeting these proteins has been proven to be a safe and effectual approach to reduce risk of coronary disease. Additionally, lowering serum LDL-C levels can reduce mortality and morbidity of cardiovascular diseases in both primary and secondary prevention (24, 25). Consistent with this result, our model also showed that LDL-C levels were the important feature in CAD risk assessment.

Large triglyceride-rich lipoprotein particles including chylomicrons (CM) and very low-density lipoprotein (VLDL) particles pass through the arterial wall via transcytosis in specialized vesicles instead of direct penetration. These particles can be swallowed by arterial macrophages, with enormous cholesterol depositing and foam cell formation in coronary arteries (26). Therefore, elevated blood triglyceride levels were associated with the development of CAD by directly participating in atherosclerotic plaque formation and progression (27). However, the relationship between increased plasma triglyceride levels and cardiovascular disease were controversial in epidemiological studies. A large meta-analysis comprising 10,158 CAD patients from 262,525 individuals in 29 prospective studies suggested a modestly significant correlation between triglyceride levels and CAD risk (28). In contrast, other studies didn't find significance after multivariable adjustments for smoking, hypertension, diabetes, BMI, and glucose levels. Meanwhile, some of these studies also implied that even a little elevated triglyceride levels were correlated with high risk of recurrent CVD events in patients receiving stain treatment and should be regarded as a useful risk indicator (29). Moreover, genetic studies have demonstrated that increased blood triglyceride levels were causally associated with high CAD risk (30). In addition, a Mendelian randomization study revealed that genetically decreased non-fasting plasma triglyceride concentrations could reduce all-cause mortality (31). In accord with these reports, our model hinted that blood triglyceride levels were helpful for CAD risk prediction.

Aside from HDL-C, LDL-C, and triglyceride levels, other variables such as BMI, TC, DBP, SBP, FBG, age, hypertension, smoking, sex, T2DM, hyperlipidemia, and drinking are of relative importance in stratifying a patient's risk. Age is the most important factor associated with the progression of CAD, as well as death when coronary atherosclerosis occurs (32). Previous studies have demonstrated that there is a conspicuous sex difference in CAD incidence and mortality (33, 34). In general, men develop CAD earlier than women (35). Dietary cholesterol could raise the concentration of serum total cholesterol, which was associated with a high risk of cardiovascular disease (36). Obesity has been shown to be an usual cause of cardiovascular mortality in the developed countries (37). Abdominal visceral with an excess fat overload can result in atherosclerotic disease (37). Dysregulation of endocrine factors originating from adipocyte in overnutrition has been presumed to be implicated in the progression of atherosclerosis (38). Hypertension was pathologically related with CAD and arterial hypertension could aggravate CAD (39). Furthermore, hypertension was also often correlated with other risk factors of CAD, such as dyslipidemia and insulin resistance (40). Diabetes was reported to frequently correlated with high levels of triglyceride and low levels of HDL-C (41). Smoking could induce endothelial exposure and platelet adhering to subintimal layer, thus increasing lipoprotein particle penetration and proliferation of smooth muscle cells (SMCs) (42). Meanwhile, the cardiovascular system is sensitive to the toxic effects of alcohol. High-dose alcohol drinking could induce extensive coronary arterial damage and increase the risk of developing CAD (43).

The Framingham risk score is a well-known prediction algorithm that has been widely applied to evaluate CAD risk in different populations including Chinese (44, 45). However, since the risk equation was developed in 1976 and more than 99% participants are of European descent, it is necessary to reconfirm the predictive values of traditional risk factors for Chinese due to the intrinsic discrepancy of diet and life style, social environment and genetic predisposition. Furthermore, rapidly increasing per-capita income, westernization of lifestyle, an aging population and longer life spans contributed to conspicuous changes in the CVD epidemics and risk factors pattern in China during the past decade (46). Therefore, an evolutionary CAD risk appraisal tool developed from recent information of Chinese population would be better generalized. Some recent studies utilized nomogram to assess CAD risks based on the results of multivariate logistic regression or Cox proportional hazard regression (47–50). Albeit these studies provided powerful clinical benefits, these models had the inherent drawback that the algorithm is sensitive to multicollinearity and missing values. Random forest is an ensemble classifier which applies lots of decision trees to the dataset and integrates results from all the trees by taking a majority vote. It can ameliorate prediction accuracy without considerably increasing the calculation amount. Our study established and validated a Random Forest model, which shows favorable predictive capability and clinical application value.

Some possible limitations in our study should be emphasized. First, this is a retrospective study, some potential inherent biases cannot be ignored and causal inference is limited. Second, we only took 15 CAD traditional risk factors into account, future studies with more variables including individual genetic information are necessitated to further confirm our results. Finally, this was a three-center study of only Chinese population from two provinces, which may restrict its generalizability. Therefore, future prospective multicenter studies from other areas of China are required to validate the findings of our study.

Collectively, an easy-to-use tool that combined 15 indexes to assess the CAD risk was constructed and validated using Random Forest algorithm, which showed favorable predictive capability (http://45.32.120.149:3000/randomforest). Our model is extremely valuable for clinical practice, which will be helpful for the primary prevention and management of CAD patients.

## Data Availability Statement

The raw data supporting the conclusions of this article will be made available by the authors, without undue reservation.

## Ethics Statement

The studies involving human participants were reviewed and approved by the Ethics Committees of Wuhan Asia Heart Hospital, Zhongnan Hospital of Wuhan University, and Shandong Provincial Hospital. The patients/participants provided their written informed consent to participate in this study.

## Author Contributions

XG, BL, YX, XZ, and ZL collected clinical information and laboratory data. CW and BJ analyzed the data. CW and YZ generated the figures and wrote the manuscript. FZ designed and supervised this study and revised the manuscript. All authors read and approved the final manuscript.

## Conflict of Interest

The authors declare that the research was conducted in the absence of any commercial or financial relationships that could be construed as a potential conflict of interest.

## References

[1] Zhu KF Wang YM Zhu JZ Zhou QY Wang NF. National prevalence of coronary heart disease and its relationship with human development index: a systematic review. Eur J Prev Cardiol. (2016) 23:530–43. 10.1177/2047487315587402 25976715

[2] Roth GA Abate D Abate KH Abay SM Abbafati C Abbasi N. Global, regional, and national age-sex-specific mortality for 282 causes of death in 195 countries and territories, 1980-2017: a systematic analysis for the Global Burden of Disease Study 2017. Lancet. (2018) 392:1736–88. 10.1016/s0140-6736(18)32203-7 30496103 PMC6227606

[3] Hansson GK. Inflammation, atherosclerosis, and coronary artery disease. N Engl J Med. (2005) 352:1685–95. 10.1056/NEJMra043430 15843671

[4] Willer CJ Sanna S Jackson AU Scuteri A Bonnycastle LL Clarke R. Newly identified loci that influence lipid concentrations and risk of coronary artery disease. Nat Genet. (2008) 40:161–9. 10.1038/ng.76 18193043 PMC5206900

[5] Levine GN Bates ER Blankenship JC Bailey SR Bittl JA Cercek B 2015 ACC/AHA/SCAI focused update on primary percutaneous coronary intervention for patients with ST-elevation myocardial infarction: an update of the 2011 ACCF/AHA/SCAI guideline for percutaneous coronary intervention and the 2013 ACCF/AHA guideline for the management of ST-elevation myocardial infarction: a report of the American College of Cardiology/American Heart Association Task Force on Clinical Practice Guidelines and the Society for Cardiovascular Angiography and Interventions. Circulation. (2016) 133:1135–47. 10.1161/cir.0000000000000336 26490017

[6] de Gonzalo-Calvo D Vilades D Martínez-Camblor P Vea À Nasarre L Sanchez Vega J Circulating microRNAs in suspected stable coronary artery disease: a coronary computed tomography angiography study. J Intern Med. (2019) 286:341–55. 10.1111/joim.12921 31141242

[7] Messerli M Panadero AL Giannopoulos AA Schwyzer M Benz DC Gräni C. Enhanced radiation exposure associated with anterior-posterior x-ray tube position in young women undergoing cardiac computed tomography. Am Heart J. (2019) 215:91–4. 10.1016/j.ahj.2019.05.006 31295633

[8] Khera AV Emdin CA Drake I Natarajan P Bick AG Cook NR. Genetic risk, adherence to a healthy lifestyle, and coronary disease. N Engl J Med. (2016) 375:2349–58. 10.1056/NEJMoa1605086 27959714 PMC5338864

[9] Kuulasmaa K Tunstall-Pedoe H Dobson A Fortmann S Sans S Tolonen H. Estimation of contribution of changes in classic risk factors to trends in coronary-event rates across the WHO MONICA Project populations. Lancet. (2000) 355:675–87. 10.1016/s0140-6736(99)11180-2 10703799

[10] Law MR Wald NJ Rudnicka AR. Quantifying effect of statins on low density lipoprotein cholesterol, ischaemic heart disease, and stroke: systematic review and meta-analysis. BMJ. (2003) 326:1423. 10.1136/bmj.326.7404.1423 12829554 PMC162260

[11] Clarke R Emberson JR Parish S Palmer A Shipley M Linksted P. Cholesterol fractions and apolipoproteins as risk factors for heart disease mortality in older men. Arch Intern Med. (2007) 167:1373–8. 10.1001/archinte.167.13.1373 17620530

[12] Breslow JL. Genetics of lipoprotein abnormalities associated with coronary artery disease susceptibility. Annu Rev Genet. (2000) 34:233–54. 10.1146/annurev.genet.34.1.233 11092828

[13] Castelli WP. Cholesterol and lipids in the risk of coronary artery disease–the Framingham Heart Study. Can J Cardiol. (1988) 4(Suppl A):5–10a 3179802

[14] Di Angelantonio E Sarwar N Perry P Kaptoge S Ray KK Thompson A. Major lipids, apolipoproteins, and risk of vascular disease. JAMA. (2009) 302:1993–2000. 10.1001/jama.2009.1619 19903920 PMC3284229

[15] Navab M Reddy ST Van Lenten BJ Fogelman AM. HDL and cardiovascular disease: atherogenic and atheroprotective mechanisms. Nat Rev Cardiol. (2011) 8:222–32. 10.1038/nrcardio.2010.222 21304474

[16] Besler C Heinrich K Rohrer L Doerries C Riwanto M Shih DM. Mechanisms underlying adverse effects of HDL on eNOS-activating pathways in patients with coronary artery disease. J Clin Invest. (2011) 121:2693–708. 10.1172/jci42946 21701070 PMC3223817

[17] Huang Y Wu Z Riwanto M Gao S Levison BS Gu X. Myeloperoxidase, paraoxonase-1, and HDL form a functional ternary complex. J Clin Invest. (2013) 123:3815–28. 10.1172/jci67478 23908111 PMC3754253

[18] Voight BF Peloso GM Orho-Melander M Frikke-Schmidt R Barbalic M Jensen MK. Plasma HDL cholesterol and risk of myocardial infarction: a mendelian randomisation study. Lancet. (2012) 380:572–80. 10.1016/s0140-6736(12)60312-2 22607825 PMC3419820

[19] Schwartz GG Olsson AG Abt M Ballantyne CM Barter PJ Brumm J. Effects of dalcetrapib in patients with a recent acute coronary syndrome. N Engl J Med. (2012) 367:2089–99. 10.1056/NEJMoa1206797 23126252

[20] Landray MJ Haynes R Hopewell JC Parish S Aung T Tomson J. Effects of extended-release niacin with laropiprant in high-risk patients. N Engl J Med. (2014) 371:203–12. 10.1056/NEJMoa1300955 25014686

[21] Cannon CP Blazing MA Giugliano RP McCagg A White JA Theroux P. Ezetimibe added to statin therapy after acute coronary syndromes. N Engl J Med. (2015) 372:2387–97. 10.1056/NEJMoa1410489 26039521

[22] Collins R Reith C Emberson J Armitage J Baigent C Blackwell L. Interpretation of the evidence for the efficacy and safety of statin therapy. Lancet. (2016) 388:2532–61. 10.1016/s0140-6736(16)31357-5 27616593

[23] Sabatine MS Giugliano RP Keech AC Honarpour N Wiviott SD Murphy SA. Evolocumab and clinical outcomes in patients with cardiovascular disease. N Engl J Med. (2017) 376:1713–22. 10.1056/NEJMoa1615664 28304224

[24] Graham I Cooney MT Bradley D Dudina A Reiner Z Dyslipidemias in the prevention of cardiovascular disease: risks and causality. Curr Cardiol Rep. (2012) 14:709–20. 10.1007/s11886-012-0313-7 22965836

[25] Reiner Ž Statins in the primary prevention of cardiovascular disease. Nat Rev Cardiol. (2013) 10:453–64. 10.1038/nrcardio.2013.80 23736519

[26] Goldstein JL Ho YK Brown MS Innerarity TL Mahley RW. Cholesteryl ester accumulation in macrophages resulting from receptor-mediated uptake and degradation of hypercholesterolemic canine beta-very low density lipoproteins. J Biol Chem. (1980) 255:1839–48. 7354064

[27] Alaupovic P Mack WJ Knight-Gibson C Hodis HN. The role of triglyceride-rich lipoprotein families in the progression of atherosclerotic lesions as determined by sequential coronary angiography from a controlled clinical trial. Arterioscler Thromb Vasc Biol. (1997) 17:715–22. 10.1161/01.atv.17.4.715 9108785

[28] Sarwar N Danesh J Eiriksdottir G Sigurdsson G Wareham N Bingham S. Triglycerides and the risk of coronary heart disease: 10,158 incident cases among 262,525 participants in 29 Western prospective studies. Circulation. (2007) 115:450–8. 10.1161/circulationaha.106.637793 17190864

[29] Faergeman O Holme I Fayyad R Bhatia S Grundy SM Kastelein JJ. Plasma triglycerides and cardiovascular events in the Treating to New Targets and Incremental Decrease in End-Points through Aggressive Lipid Lowering trials of statins in patients with coronary artery disease. Am J Cardiol. (2009) 104:459–63. 10.1016/j.amjcard.2009.04.008 19660594

[30] Do R Willer CJ Schmidt EM Sengupta S Gao C Peloso GM. Common variants associated with plasma triglycerides and risk for coronary artery disease. Nat Genet. (2013) 45:1345–52. 10.1038/ng.2795 24097064 PMC3904346

[31] Thomsen M Varbo A Tybjærg-Hansen A Nordestgaard BG. Low nonfasting triglycerides and reduced all-cause mortality: a mendelian randomization study. Clin Chem. (2014) 60:737–46. 10.1373/clinchem.2013.219881 24436475

[32] Goff DC Jr Lloyd-Jones DM Bennett G Coady S D'Agostino RB Gibbons R 2013 ACC/AHA guideline on the assessment of cardiovascular risk: a report of the American College of Cardiology/American Heart Association Task Force on Practice Guidelines. Circulation. (2014). 129(25 Suppl. 2), S49–73. 10.1161/01.cir.0000437741.48606.98 24222018

[33] Jousilahti P Vartiainen E Tuomilehto J Puska P. Sex, age, cardiovascular risk factors, and coronary heart disease: a prospective follow-up study of 14 786 middle-aged men and women in Finland. Circulation. (1999) 99:1165–72. 10.1161/01.cir.99.9.1165 10069784

[34] Rosamond W Flegal K Friday G Furie K Go A Greenlund K. Heart disease and stroke statistics−2007 update: a report from the American Heart Association Statistics Committee and Stroke Statistics Subcommittee. Circulation. (2007) 115:e69–171. 10.1161/circulationaha.106.179918 17194875

[35] Anand SS Islam S Rosengren A Franzosi MG Steyn K Yusufali AH. Risk factors for myocardial infarction in women and men: insights from the INTERHEART study. Eur Heart J. (2008) 29:932–40. 10.1093/eurheartj/ehn018 18334475

[36] Berger S Raman G Vishwanathan R Jacques PF Johnson EJ. Dietary cholesterol and cardiovascular disease: a systematic review and meta-analysis. Am J Clin Nutr. (2015) 102:276–94. 10.3945/ajcn.114.100305 26109578

[37] Matsuzawa Y Nakamura T Shimomura I Kotani K Visceral fat accumulation and cardiovascular disease. Obes Res. (1995) 3(Suppl. 5):645–7s. 10.1002/j.1550-8528.1995.tb00481.x 8653544

[38] Kumada M Kihara S Sumitsuji S Kawamoto T Matsumoto S Ouchi N. Association of hypoadiponectinemia with coronary artery disease in men. Arterioscler Thromb Vasc Biol. (2003) 23:85–9. 10.1161/01.atv.0000048856.22331.50 12524229

[39] Chobanian AV Alexander RW. Exacerbation of atherosclerosis by hypertension. Potential mechanisms and clinical implications. Arch Intern Med. (1996) 156:1952–1956. 8823148

[40] DeFronzo RA Ferrannini E. Insulin resistance. A multifaceted syndrome responsible for NIDDM, obesity, hypertension, dyslipidemia, and atherosclerotic cardiovascular disease. Diabetes Care. (1991) 14:173–94. 10.2337/diacare.14.3.173 2044434

[41] Haffner SM. Diabetes, hyperlipidemia, and coronary artery disease. Am J Cardiol. (1999) 83:17–21f. 10.1016/s0002-9149(99)00213-1 10357570

[42] Malakar AK Choudhury D Halder B Paul P Uddin A Chakraborty S. A review on coronary artery disease, its risk factors, and therapeutics. J Cell Physiol. (2019) 234:16812–23. 10.1002/jcp.28350 30790284

[43] Kim MK Shin J Kweon SS Shin DH Lee YH Chun BY. Harmful and beneficial relationships between alcohol consumption and subclinical atherosclerosis. Nutr Metab Cardiovasc Dis. (2014) 24:767–76. 10.1016/j.numecd.2014.02.004 24694837

[44] Wilson PW D'Agostino RB Levy D Belanger AM Silbershatz H Kannel WB. Prediction of coronary heart disease using risk factor categories. Circulation. (1998) 97:1837–47. 10.1161/01.cir.97.18.1837 9603539

[45] Chen G Levy D. Contributions of the framingham heart study to the epidemiology of coronary heart disease. JAMA Cardiol. (2016) 1:825–30. 10.1001/jamacardio.2016.2050 27464224 PMC9316391

[46] Cheng J Zhao D Zeng Z Critchley JA Liu J Wang W. The impact of demographic and risk factor changes on coronary heart disease deaths in Beijing, 1999-2010. BMC Public Health. (2009) 9:30. 10.1186/1471-2458-9-30 19159492 PMC2637858

[47] Naoum C Berman DS Ahmadi A Blanke P Gransar H Narula J. Predictive value of age- and sex-specific nomograms of global plaque burden on coronary computed tomography angiography for major cardiac events. Circ Cardiovasc Imaging. (2017) 10:e004896. 10.1161/circimaging.116.004896 28292858

[48] Hartaigh BÓ Gransar H Callister T Shaw LJ Schulman-Marcus J Stuijfzand WJ. Development and validation of a simple-to-use nomogram for predicting 5-, 10-, and 15-year survival in asymptomatic adults undergoing coronary artery calcium scoring. JACC Cardiovasc Imaging. (2018) 11:450–8. 10.1016/j.jcmg.2017.03.018 28624402 PMC5723248

[49] Wu N Chen X Li M Qu X Li Y Xie W. Predicting obstructive coronary artery disease using carotid ultrasound parameters: a nomogram from a large real-world clinical data. Eur J Clin Invest. (2018) 48:e12956. 10.1111/eci.12956 29782650

[50] Huang S Xie X Sun Y Zhang T Cai Y Xu X Development of a nomogram that predicts the risk for coronary atherosclerotic heart disease. Aging. (2020) 12:9427–39. 10.18632/aging.103216 32421687 PMC7288976

